# A pilot evaluation of the Baby Social ABCs caregiver-mediated intervention for 6–15-month-olds with early signs of autism—feasibility, acceptability, and preliminary evidence

**DOI:** 10.3389/frcha.2025.1689781

**Published:** 2025-10-24

**Authors:** E. Dowds, S. MacWilliam, A. Solish, S. Osten, L. Zwaigenbaum, I. M. Smith, J. A. Brian

**Affiliations:** ^1^ Autism Research Centre (ARC), Holland Bloorview Kids Rehab, Toronto, ON, Canada; ^2^ Autism Research Centre, IWK Health, Halifax, NS, Canada; ^3^ Autism Research Centre, Glenrose Rehabilitation Hospital, Edmonton, AL, Canada

**Keywords:** autism, infants, caregiver-mediated, early intervention, caregiver coaching

## Abstract

**Background:**

Autism spectrum disorder (autism) is a neurodevelopmental condition with a high prevalence of approximately 1 in 50 children. Early intervention can support long-term outcomes. Caregiver-mediated interventions (CMIs) are evidence-based and appropriate for toddlers with autism or early social communication challenges. The Social ABCs, one such CMI, is supported by robust evidence. Originally developed for toddlers (12–42 months), it shows potential for supporting social communication development even earlier, i.e., for infants with early signs of autism. The current project adapted the toddler Social ABCs for use with infants (aged 6–15 months) showing early signs of autism or with a confirmed diagnosis. This paper describes the development, acceptability, feasibility, and preliminary outcomes for the Baby Social ABCs.

**Methods:**

Nine infants (aged 6–14 months) participated. Families either self-referred or were referred by community clinicians and were eligible based on age and clinician and/or parent concerns about social communication and/or behavioral differences. Each infant and one of their primary caregivers participated in the 12-week Baby Social ABCs intervention online via Zoom for Healthcare.

**Results:**

Caregiver implementation fidelity increased significantly, along with infant responsivity and social communication behaviors (social orienting, shared smiling, and gesturing). The caregivers reported high satisfaction with the coaching approach, session structure, and curriculum.

**Discussion:**

This pilot study demonstrated the feasibility and acceptability of the Baby Social ABCs as a novel CMI for infants with signs of emerging autism and showed promising effects on the caregivers’ fidelity and the infants’ social communication and engagement. Future research should consider the optimal timing (or personalized “fit”) for families to access such support to better understand the type and intensity of pre-diagnostic care that best meets families’ diverse needs.

## 1 Introduction

Autism spectrum disorder (ASD; autism) is a neurodevelopmental condition with a high prevalence of approximately 1 in 50 children in Canada between the ages of 1 and 17 years (1). Access to early intervention and support for young autistic children is a health priority (2) as delays are associated with poorer long-term outcomes (3–5), which can be mitigated by timely access to early intervention (6, 43). Very early intervention leverages the brain's plasticity, optimizing long-term developmental outcomes in autistic children (2, 6–8). The inclusion of caregivers in early intervention (i.e., caregiver-mediated approaches) is thought to capitalize on natural caregiver–child interactions and increase the child's treatment dosage while also supporting families (9) at a key point in their parenting journey when they may be particularly vulnerable to parenting-related stressors associated with feelings of inefficacy (10).

Caregiver-mediated early interventions have been developed to address the need for early, resource-efficient treatment options and have been rigorously studied, yielding a substantial evidence base (11, 12). While such interventions are well-studied in toddlerhood and early childhood [i.e., 12–42 months (11, 13, 14)], research specifically targeting infants under 12 months remains limited, with most studies identifying their youngest participants as being “under 24 months” (15) and limited focus on those nearest to 12 months.

Zhao et al. (14) conducted a meta-analysis of parent-mediated interventions (hereafter, caregiver-mediated interventions; CMIs) for autism evaluated through randomized controlled trials (RCTs) in children under 3 years old. Overall, the interventions showed small but significant improvements in toddler adaptive skills, parent responsiveness, and parent–child interactions and reduced social communication challenges and autism symptoms. However, only five studies included participants as young as 12 months of age (16–19, 44), some of which included participants up to 36 months, making it challenging to determine how interventions uniquely impact infants under 12 months compared to older infants and toddlers.

A meta-analysis by Hampton and Rodriguez (20) examined interventions for infants and toddlers at an elevated likelihood for or showing early signs of autism, with only four studies including infants younger than 12 months (21–25). These interventions largely focused on increasing children's responsiveness to caregivers, joint attention behaviors, play, and social communication through a combination of coaching, video review, modelling, and education sessions. Overall, studies show a positive association between caregiver fidelity (i.e., use of the strategies as intended) and child outcomes, with some studies showing short-term changes and others only showing changes after a follow-up period [e.g., (21, 22, 45)]. Given the variability across studies and participants, the authors highlighted the importance of taking an individualized approach to intervention. A call has also been made for future research to identify the effective components of interventions for infants under 15 months and their families (26).

The Social ABCs is a caregiver-mediated intervention originally developed for toddlers aged from 12 to 42 months with autism or related social communication and/or behavioural features (27, 28). The Social ABCs has been shown to improve early social communication in toddlers with autism or early signs thereof, as demonstrated in a cross-site RCT (28), a large-scale community effectiveness study (29), and a large pilot evaluation of an abbreviated group-based virtual model (30). In addition to child-level gains, caregivers also made significant gains in fidelity of implementing intervention strategies and reported feeling empowered and satisfied with the program. Following the group-based model, caregivers also reported reduced parenting stress, particularly when accessing the program in person (30).

The Social ABCs program was originally developed for use with toddlers as young as 12 months (though most published studies have not included participants <14 months), and as such, it shows potential for supporting social communication development in even younger infants with early signs of autism. Specifically, the approach used (i.e., positive, in-the-moment, supportive caregiver coaching) makes it well-suited for caregivers who require a sensitive and individualized approach when they are already aware of emerging concerns in the earliest months of their baby's development (31) and caregiver mental wellbeing may be particularly vulnerable (10).

The objective of the current project was to adapt the toddler Social ABCs for use with infants aged between 6 and 15 months showing early signs of autism or with a confirmed diagnosis. In this article, we describe the development of the Baby Social ABCs, outline key adaptations to the standard (toddler) model, and examine the acceptability, feasibility, and preliminary outcomes related to both caregiver implementation and infant development. Specifically, we aimed to assess the following: feasibility (recruitment, retention, and session attendance), acceptability of the program to caregivers (satisfaction), caregiver learning outcomes (fidelity of implementation), and infant response to the intervention (focusing on the caregiver–child interactions described below).

## 2 Methods

### 2.1 Participants

Nine infants participated, ranging in age from 6 to 14 months (*M* = 9.56 months; *SD* = 2.30). The sample consisted of infants aged 6 months (*n* = 1), 7 months (*n* = 1), 9 months (*n* = 2), 10 months (*n* = 3), 11 months (*n* = 1), and 14 months (*n* = 1). Pre-intervention scores on the Communication and Symbolic Behaviour Scales: Infant-Toddler Checklist (CSBS-ITC) and the Parent-Rated Observation of Communication, Emotions, and Social Skills (PROCESS) questionnaire were variable across the babies—see Table 1 for means, SD, and ranges, and Table 2 for other participant and family characteristics.

**Table 1:** Baseline and session data.

Variable	Mean	Standard deviation (SD)	Minimum value	Maximum value
Infant age (months)	9.56	2.297	6.00	14.00
CSBS-ITC^a^	10.83	5.076	5.00	31.00
PROCESS^b^	22.00	8.786	11.00	36.00
Parenting self-efficacy	58.60	8.849	47.00	72.00

^a^ CSBS-ITC, Communication and Symbolic Behavior Scales: Infant-Toddler Checklist (33).

^b^ PROCESS, Parent-Reported Observation of Communication, Emotion, and Social Skills [previously, APSI; (32)].

**Table 2:** Participants’ descriptive data.

Participant variable	Number of participants (*n*)	Percentage of participants (%)
Sex		
Female	4	44.4
Male	5	55.6
Ethnicity		
Middle Eastern	1	11.1
East Asian	1	11.1
White	7	77.8
Sibling with autism		
No	5	55.6
Yes	4	44.4
Parent coached		
Father	2	22.2
Mother	7	77.8
Did the parent have concerns?		
ASD diagnosis given	1	11.1
No concerns reported	2	22.2
Yes, concerned	6	66.7
Parent education		
BA/BSc	3	33.3
Graduate/ MBA/MD	6	66.6

### 2.2 Procedure

Participants either self-referred after hearing about the program or were referred by community clinicians (e.g., pediatricians, early interventionists, and speech-language pathologists) who identified concerns about early features of autism and provided the families with information about the Baby Social ABCs study. Eligibility required the caregivers to be aware of these concerns and willing to participate in a research intervention. Interested families contacted the study coordinator, who provided study details and obtained informed consent.

The participants completed a Parent Concerns interview with the study coordinator and were eligible based on the age of the baby (6–15 months) and clinician and/or parent concerns about social communication and/or behavioral differences, and a strong desire from the caregivers to participate in and learn about the Baby Social ABCs. All potential participants were assessed using a validated parent-report measure of early autistic features (described below), but elevated scores were not required for enrolment. All the infants were born at >36 weeks’ gestation, with no known medical complexities or genetic conditions, as per the inclusion criteria. Each infant and one of their primary caregivers participated in the 12-week Baby Social ABCs intervention. The intervention was delivered online via Zoom for Healthcare with the participants enrolled through the Autism Research Centre at Holland Bloorview Kids Rehabilitation Hospital in Toronto, Ontario, Canada.

### 2.3 Measures

#### 2.3.1 Questionnaires

Demographic information was collected using a family profile form completed by the caregiver who was selected by the family to receive the coaching (hereafter, “coached caregiver”). The coached caregiver also completed a “Parent Concerns” interview (31) and the following four questionnaires: (1) The PROCESS© [formerly APSI (32, 46)] is a 26-item forced-choice (“yes, sometimes, or no”) questionnaire used to characterize autism-related features in infants between 6 and 24 months old. Higher scores indicate the presence of more frequent and/or marked autism-related behaviour. The sensitivity and specificity for total scores are as follows: 0.67 and 0.86 at 6 months (cutoff of 15), 0.59 and 0.72 at 12 months (cutoff of 10), and 0.65 and 0.72 at 18 months (cutoff of 9). Internal consistency ranges from fair to excellent for the current study's age range (0.77, 0.90, 0.83, 0.89 at 6, 9, 12, and 15 months, respectively), ascertained in a sample of toddlers with elevated ASD-likelihood (i.e., younger siblings of autistic children) who themselves received an autism diagnosis at 3 years of age (32). (2) CSBS-ITC (33) is a 24-item parent-rated questionnaire that was developed to capture social communication development in toddlers aged 6–24 months, with excellent sensitivity for ASD prediction [i.e., 93%; (33)]. Higher scores on the CSBS-ITC reflect greater developmental progress, with the following total scores identified as cutoffs for concern across the ages in the current study: total score ≤12 at age 6 months, ≤13 at 7 months, ≤17 at 9 months, ≤22 at 10 months, ≤24 at 11 months, and ≤32 at 14 months (33). The PROCESS and CSBS-ITC questionnaires were both completed at baseline to characterize parent-reported social communication challenges, but no specific cutoff criteria were used to determine eligibility. (3) The caregivers also completed a 21-item self-report self-efficacy measure that was developed for and has been used in related studies (27), with a maximum score of 105. Previous studies on the toddler Social ABCs yielded mean self-efficacy scores of 59–61 at baseline (28). (4) Finally, a satisfaction questionnaire, adapted from one used in related studies (27, 28), was completed at the end of the program. The satisfaction questionnaire comprised seven questions about satisfaction with the program, the materials, and the coaching, scored on a scale of 1 (not at all) to 5 (extremely satisfied), yielding a maximum score of 35. Three additional questions asked about the appropriateness of training duration, number of sessions, and session length (rated as follows: 1: “too short”; 2: “just right”; or 3: “too long”). The caregivers were invited to add other comments in a free-text box at the end of the questionnaire.

As per our previous work with older toddlers (27, 28), the participating families were also invited to provide informal feedback during or following each session regarding their own and their baby's behaviour and perceived response to the intervention. This written and verbal feedback was used, iteratively, to inform adaptations, particularly as they related to the number of sessions, session length, and coaching approach.

#### 2.3.2 Video-coded data

Video recordings of the caregiver–infant interactions were collected at baseline and post-intervention and coded to measure caregiver fidelity of implementation and various infant and dyad-related behaviors (defined below), using a coding scheme adapted from studies on the standard Social ABCs (Table 3).

**Table 3:** Coding definitions.

Coding terms	Positive (+) score
Caregiver strategy	
Child choice	The caregiver creates opportunities in the context of the child’s interest/motivation
Child attending	The caregiver presents an opportunity when the child is looking at the object/activity/caregiver
Shared control	The caregiver holds/blocks/pauses access to an object/activity prior to presenting an opportunity
Clear opportunity	A one-word model prompt that is appropriate to an activity/object, presented in a neutral tone
Pace	Opportunities are presented in an appropriate balance with play (e.g., child has time to play with the toy between opportunities)
Contingent	a) The caregiver reinforces a directed, intentional response within 2–3 s (immediate). b) Reinforcement is natural (directly tied to the opportunity). c) The caregiver does not reinforce in the absence of a directed response (and continues to prompt if appropriate)
Contingent on attempts	Any intentional goal-directed attempt to respond to an opportunity is reinforced
Infant/dyad behavior	
Shared positive emotion	Simultaneous smiles from the baby and caregiver, including smiles directed at each other and mutual smiles aimed at an activity/toy/event
Social orienting	Visual check-ins from the baby to the caregiver
Responsivity	The baby’s responses (e.g., vocalizations or gestures) to caregiver-provided communication opportunities, including model prompts (MP), questions, leading prompts and time delays • Responsivity-MP: the baby’s responses to caregiver-provided model prompts (intentional one-word verbal cues) Response types: • Vocal: baby responds to the caregiver with a directed vocalization • Gestural: baby responds to the caregiver with a directed gesture (e.g., reaching or pointing) • Vocal + gestural: baby responds to the caregiver with a combined vocalization plus gesture

To establish the coding rules, a primary rater and a secondary rater jointly coded four randomly selected videos [two from baseline (BL) and two post-intervention (PI)]. After reaching an agreement on the coding rules, the primary rater independently coded the remaining videos. Additionally, 20% of the videos (specifically, two BL and two PI videos) were coded by the secondary rater to assess the inter-rater reliability. All videos were coded blind to the study phase.

#### 2.3.3 Development of coding reliability

Following the methods outlined by Koegel and Koegel (47), parent fidelity was assessed through video analysis using continuous interval coding, which consisted of ten 1-min intervals. Each interval was categorized as either correct implementation (+) or incorrect implementation/not used (−) for each of the seven techniques outlined in Table 3. These techniques included (1) child choice, (2) child attending (to caregiver or activity/object), (3) shared control, (4) clear opportunity, (5) pace, (6) contingent reinforcement, and (7) reinforcement of attempts. The fidelity of implementation score was calculated as the total percentage of intervals across all seven techniques, during which the parents effectively demonstrated the appropriate use of the techniques. The inter-rater reliability averaged 92% agreement (range of 90%–94%).

The following infant and dyadic behaviors were also coded from the video segments. (1) Infant responsivity to caregiver-provided communication opportunities. Communication opportunities included one-word model prompts (MP), questions, leading prompts, and time delays initiated by the caregiver. A response from the infant was defined as any vocalization, gesture, or combination of both that occurred immediately following the caregiver's communication opportunity. Responsivity was calculated as a percentage using the following formula: Responsivity (%) = (number of the infant's responses to caregiver's language opportunities ÷ total number of caregiver's language opportunities) × 100. We also coded (2) infant social orienting (i.e., child-initiated visual check-ins with the caregiver, reported as total counts) and (3) shared smiling between infant and caregiver (coded as present/absent per 10 s segment). See Table 3 for definitions.

#### 2.3.4 Treatment approach: Baby Social ABCs

The approach used in the current paper entailed an adaptation of the Social ABCs program for older toddlers (27, 28). The model used here (Baby Social ABCs) includes refinements based on knowledge of infant development, with learning priorities identified as essential for early intervention in infants with early features of autism, namely, early attentional control, emotion regulation, social orienting/approach, and communication development (7). These four domains served as the foundation for the adapted Baby Social ABCs, which aimed to provide caregivers with a skill set that was intended to increase the clarity of their social communication cues to their baby, while supporting the caregivers’ sensitivity to cues from the baby that may be subtle, ambiguous, or otherwise difficult to interpret.

The Baby Social ABCs intervention entailed 15 online coaching sessions over a 12-week period. Each session lasted 45–60 min, with the duration adjusted based on family availability and feasibility (infant's alertness, regulation, nap schedule, feeding needs, and caregiver-infant enjoyment). Two teaching approaches were used, namely, direct coaching and didactic instruction.

#### 2.3.5 Didactic instruction

**Figure 1:**
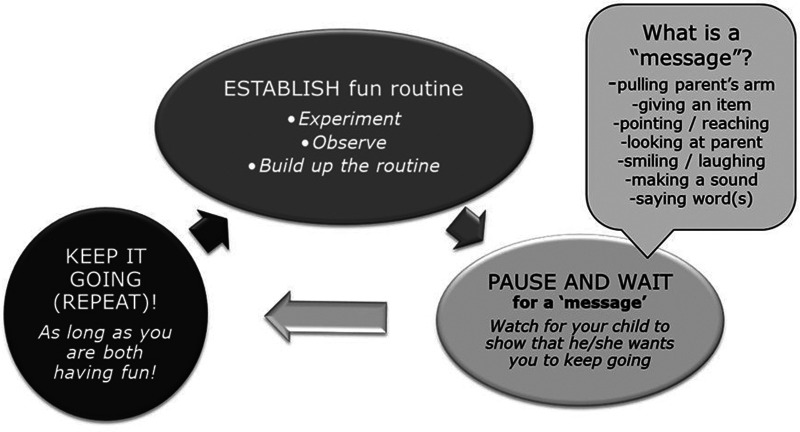
“EPK” visual framework for engaging babies in the infant Social ABCs.

In addition to direct, *in vivo* coaching (described below), all the caregivers received didactic instruction. Didactic content was presented in 20-min blocks within three of the first coaching sessions (using a “While You Wait” presentation developed by co-author JB for caregivers of infants and toddlers awaiting autism assessment and access to other services). The focus was on teaching caregivers how to build a social-communication routine using the “Epic (EPK)” framework (E: Establish a routine, P: Pause and wait for a message, K: Keep it going as long as the baby is engaged and happy). These EPK routines served as occasions for caregivers to create opportunities for the baby to engage in intentional directed communication (Figure 1).

#### 2.3.6 Direct coaching

Most of the learning took place via synchronous virtual caregiver coaching [as described in the standard Social ABCs (27)] using every-day routines and play experiences within the family's daily life. The Baby Social ABCs coach (author ED) provided direct, in-the-moment instruction to the caregivers on how to create communication opportunities. The caregivers were coached to respond immediately to all directed communication attempts from the baby, including but not limited to directed gaze, body movements or gestures intended to convey a message, and any vocalization.

Additionally, the coach identified subtle cues from the baby and supported caregivers in interpreting their baby's intentions (or cues). Understanding the baby's intentions was made easier by ensuring that the dyad was positioned face-to-face, which also served as the foundation for positive emotion-sharing between the dyad, which was encouraged by coaching the caregiver to smile, behave playfully, and entice the infant's attention. The caregivers were coached to follow their infant’s natural interests and prompted to provide language opportunities based on their infant’s motivation.

Finally, the caregivers were encouraged to pause in creating social communication opportunities whenever their baby was actively attempting to engage in an emerging motor behavior, such as crawling, pulling to stand, standing without support, and in many cases, attempting to cruise or take first steps. Frequently, throughout the sessions, caregivers were coached to adjust the focus of the interaction away from social communication toward supporting motor development in order to follow the infant's lead and support development across domains in a naturalistic way, without expecting the baby to focus on multiple emerging skills simultaneously.

#### 2.3.7 Curriculum and coaching adaptations

The Baby Social ABCs model was trialled in one pre-pilot family with a 10-month-old, who worked in collaboration with the Social ABCs team to co-design, refine, and try the intervention approach. Following this phase, the remaining participants received the study version of the program with the following specific areas of focus:

1. Focus on developmental needs. This entailed coaching the caregivers to provide verbal and non-verbal communication opportunities, and to accept all directed communication acts by the baby. The caregivers were coached to notice emerging and developmental progress during the sessions. The coach provided in-the-moment narration to the caregivers (e.g., to point out when an infant was pulling to stand, attempting to walk, or beginning to reach/point).
2. Focus on the early parenting stage. The content for the caregivers was streamlined, using descriptive instead of technical terms (e.g., “I want you to hold onto the bubble-maker with both hands” vs. “get shared control of the bubbles”).
3. Focus on faces. This technique entailed helping the caregivers foster their infant's interest in faces over toys, including an emphasis on people-centered games rather than object-focused activities.
4. Focus on infant cues. The caregivers were supported in interpreting hard-to-read cues and coached to respond positively and contingently. The caregiver's body position is an essential element (i.e., remaining at the child's level, directly front-to-front, in close proximity) to facilitate the infant's ability to check in with the caregiver (i.e., look toward).
5. Focus on caregiver wellbeing. This technique entails validating the caregiver’s feelings of concern about their infant's development and listening without judgment. Coaching involves nourishing the caregiver’s feelings of confidence and competence, following the caregiver's ideas, reminding the caregiver that they know the infant best, identifying what is going well, and highlighting the infant's reactions to the caregiver’s engagement activities.
6. Focus on regulation. This involves modeling regulation (using a calm and supportive tone of voice) and providing the caregiver with strategies (e.g., “use your tone of voice, stay in the current moment without ‘rushing’ to the next activity, and use routines”). Here, the coach must notice any emerging stress in the caregiver–infant dyad and provide calm reassurance (e.g., “there's no rush, take your time”).

#### 2.3.8 Pedagogical approach with caregivers

In teaching skills to caregivers, the intention was to create and nourish satisfying interactions in the dyad (infant and caregiver). The positive coaching approach used with caregivers has played an integral role in caregiver skill building in the standard Social ABCs, and the stance of the coach is intentionally positive (rather than corrective). This approach is used to guide caregivers to use strategies that create occasions in which infants can benefit the most from caregiver cues, and caregivers can feel empowered to support their baby. The coach intentionally communicates to caregivers that their current parenting approach is not “wrong” and that they are not “causing” the social communication differences. Instead, caregivers learn that the intervention techniques can be helpful in optimizing the interaction given the different cues that their child may be exhibiting.

## 3 Results

### 3.1 Video-coded variables

**Figure 2:**
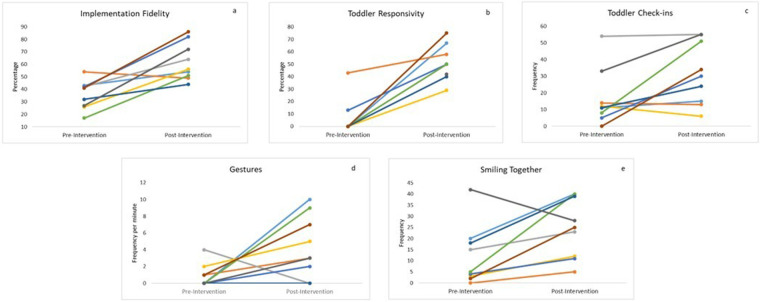
Intervention outcomes for each caregiver–infant dyad: **(a)** caregiver implementation fidelity, **(b)** toddler responsivity to caregiver's language opportunities (model prompts), **(c)** toddler check-ins, (**d**) toddler gesture use, and (**e**) caregiver and child smiling together.

Non-parametric, Wilcoxon signed-rank tests were conducted to assess changes in parent–child social communication behaviours following the Baby Social ABCs intervention (Table 4). Caregiver implementation increased significantly (*Z* = −2.55, *p* = 0.011). Infant responsivity to model prompts also increased significantly (*Z* = −2.52, *p* = 0.012), as did infant check-in behaviours (*Z* = −2.02, *p* = 0.044), and joint smiling (*Z* = −2.07, *p* = 0.038). There was a trend toward increased gesture use (*Z* = −1.83, *p* = 0.068). There were no significant effects for vocal responsivity in isolation or combined (gestures + vocal) responsivity. See Figure 2 for individual-level change in the significant video-coded variables.

**Table 4:** Video-coded data pre- and post-intervention.

Caregiver-infant behaviour	Pre-intervention	Post-intervention	Test statistic
	Mean (SD)	Mean (SD)	Z score (*p*)*
Implementation fidelity	36.00 (11.336)	62.00 (14.975)	−2.55 (.011)*
Responsivity to model prompts	7.00 (15.241)	52.375 (14.510)	−2.52 (.012)*
Check-ins	16.44 (16.742)	31.44 (18.756)	−2.02 (.044)*
Smiling together	12.11 (13.448)	24.78 (13.340)	−2.07 (.038)*
Gestures	0.89 (1.364)	4.33 (3.674)	−1.83 (.068)*
Vocal responding	2.00 (4.183)	3.44 (5.480)	−0.53 (.599)
Gestures + vocal responding combined	1.22 (1.922)	1.89 (2.205)	−.81 (.416)

* *p* < .05.

### 3.2 Association between parent change and child change

**Figure 3:**
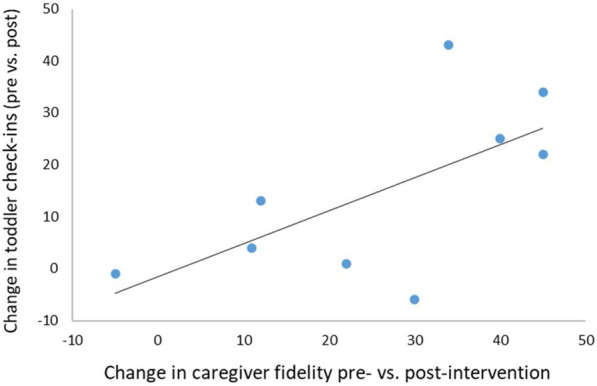
Association between the change in caregiver implementation fidelity and increased infant social orienting (rate of check-ins) to the caregiver. Spearman's rho = .653, *p* = .057.

Non-parametric correlational analyses were conducted to assess whether changes in infant behaviour were associated with the caregivers’ use of the Baby Social ABCs intervention strategies (i.e., change in implementation fidelity). The association between the change in caregiver fidelity and change in infant check-ins was the strongest (Spearman's rho = 0.653) and approached significance, (*p* = 0.057) (Figure 3). No significant associations were found between caregiver fidelity and infant vocal responsivity, smiling, gestures, or gestures combined with vocal responding.

### 3.3 Feasibility and acceptability

Eight of the nine families completed the 12-week program, with missed sessions typically rescheduled within the same week. One dyad attended 9/12 sessions prior to the mother returning to work following maternity leave. Responses regarding session length and program duration were captured on the satisfaction questionnaire. Weekly rather than twice-weekly visits were preferred by the families, who found the latter challenging, particularly those with another child already diagnosed with autism. An example of this preference was reflected by the following response on the satisfaction questionnaire:

Being a busy family, I found that 1 session per week worked well for us, instead of the 2 sessions noted in the [initial] study protocol. I think I would have felt overwhelmed with 2/week and not enough time to continue working on the skills learned in the prior session. Also, I appreciated that our sessions were kept to around 45–50 min, and [Coach] had a great sense of determining when [child] had enough for the session.

Overall caregiver satisfaction with the program was high (*M* = 89.92%; SD = 7.10). The families found the “While You Wait*”* presentation valuable and expressed interest in revisiting it throughout the program, reinforcing the need for a caregiver manual. The satisfaction scores were consistent with the open-ended responses that highlighted positive experiences with the live, in-the-moment instructional approach and being able to access the program while waiting for other services; for example:

[T]he regularity of the live coaching sessions was most helpful. Each session would reinforce the techniques while giving me new tips. And practicing live while getting feedback in real time helped me understand the concepts in my own unique context (in a way that watching a presentation, for example, could not). The regular hands-on sessions also held me accountable—after the dedicated, scheduled practice time, I’d feel re-focused and eager to try on my own again. They also served as opportunities to get clarity when questions came up…[I]n my case, my daughter began quite young around 6 months. This was a huge plus, as we were struggling to find “early intervention” that early, and me having this knowledge to apply with her at home while we wait for other services has been great.

## 4 Discussion

This pilot study demonstrated the feasibility and acceptability of the Baby Social ABCs as a novel caregiver-mediated intervention for infants with signs of emerging autism. Moreover, the intervention shows promising effects on caregivers’ fidelity of implementation and infants’ social engagement (checking in, responding, and shared smiling with caregivers).

On average, caregiver implementation fidelity increased significantly (i.e., from 36% to 62%), indicating that the parents made gains in their use of social learning strategies following the intervention. Following the intervention, mean caregiver fidelity approached the rate achieved in the abbreviated group-based virtual model for toddlers [i.e., 69.5%; (30)] but was lower than the rate reported for the original, 12-week in-person model for toddlers [i.e., >80%; (27–29)]. Notably, fidelity improved for eight families, with increases ranging from 11 to 45 percentage points. One participant experienced a small decline in fidelity, which may be attributed to a significant increase in the child's autism symptoms during participation in the program. There was considerable variability in the level of caregivers’ concerns regarding early autism indicators, with some reporting no observable concerns themselves but participating due to clinician concerns and/or in response to an awareness of increased familial autism likelihood. It is possible that our lower fidelity levels may be related to reduced parental concerns in some cases, but informal impressions from the coach, supported by positive feedback from the caregivers, suggested high levels of motivation and program “buy-in,” so this does not seem to be an adequate explanation at this point. The role of parental concern (and how it relates to program adherence) remains to be examined more fully in a larger sample.

Another possibility is that the high standard for fidelity in the in-person toddler model may have been too stringent for the Baby Social ABCs, which was delivered virtually. Alternatively, the fidelity measure may require further modifications for use in this very young age group. Specifically, some fidelity criteria may not be appropriate in the current context. For example, the current coding system penalizes caregiver fidelity scores across several fidelity categories for the use of questions as “language opportunities.” This is based on the toddler version, in which caregivers are coached to avoid excessive question-asking (particularly questions that do not necessitate a response, e.g., “You like that toy, don’t you?”). This guidance is based on the premise that such questions may confuse toddlers and inadvertently teach them not to respond to caregiver language. The appropriateness of this approach with toddlers remains an empirical question, but the use of questions (rhetorical or other) may be less problematic when working with prelinguistic infants. Caregivers may use this strategy with infants to invite social engagement (e.g., “Who's my happy baby?”) rather than as a language development strategy. Future program refinements may need to consider reclassifying the use of questions to acknowledge their use as a way to engage and foster infant attention [i.e., as an element of infant-directed speech (IDS), e.g., (34)].

Nenchva and Lew-Williams (34) recognized the role of question-asking in IDS to promote infant attention and increase engagement. Recent studies advocate incorporating IDS strategies, including the use of questions and varying vocal pitch and tone (which can accompany such questions), into early intervention protocols to enhance attentional engagement and support the development of social communication skills (34). Coaching caregivers to use questions with this style aligns with current curriculum recommendations for early autism interventions (35).

In addition to caregiver fidelity, several child-level behaviours improved from baseline to post-intervention, including responsiveness to caregivers’ strategy use. This is consistent with previous versions of the Social ABCs, but the coding in this study allowed for any form (i.e., vocal and/or gestural) of response from the baby, whereas the toddler version emphasized vocal responding. In addition to increased responses, infant-initiated social communication also increased significantly in the form of infants increasingly directing their attention to their caregiver through visual check-ins. Shared smiling and infant gestures also significantly increased, indicating enhanced shared emotional engagement and non-verbal communication.

The regression analysis showed that, as the caregivers implemented the techniques in the Baby Social ABCs, infants tended to check in with caregivers at a higher rate. The analytical power of the model was limited by the small sample size; testing this association in a larger sample is warranted.

The families found the program to be feasible and acceptable, with high attendance rates, caregivers being well prepared for coaching sessions, and evidence of caregiver learning (i.e., implementation fidelity) during the sessions. Recent research has revealed that caregivers are actively involved when they experience developmental progress in their children (36–38). In turn, a caregiver’s active involvement is directly associated with the child’s responses to the intervention strategies (37, 38), which then plays a role in shaping the caregiver's ongoing commitment to use the strategies (27, 39, 40), resulting in a positive feedback loop.

Caregiver wellbeing has also been implicated in caregiver-mediated interventions, wherein parent practice is associated with feelings of self-efficacy (41). Lee et al. (36) found that parents in the Social ABCs for toddlers reported increased feelings of wellness that were directly related to their child's progress within the program.

Support for caregivers’ concerns about their children's development and referral to continued early intervention services was a small but regular part of the Baby Social ABCs program. The recommendations involved validation of the caregivers’ concerns and ongoing encouragement for families to follow up on seeking a developmental assessment. Information on local infant and child development programs and early intervention services was also discussed during the sessions, and the families pursued these suggestions.

Grzadzinski et al. (26) considered the targets for early support for pre-symptomatic and pre-diagnostic infants, including factors related to the timing of the intervention (monitoring vs. intervention), ethical implications, and how best to design interventions that address early autism symptoms with improved long-term outcomes in mind. While some monitoring and support may benefit all babies at elevated likelihood for autism (26), the Baby Social ABCs may be most appropriate when caregiver(s) have identified some specific concerns about their baby's development or when a clinician (e.g., family doctor, speech-language pathologist, and early intervention professionals) has shared concerns with the family.

Due to the high demand in a low-resource system, it may be most feasible to offer less intensive monitoring and developmental surveillance for high likelihood babies, and present the option for a program such as Baby Social ABCs if/when autistic traits appear. Engaging in a caregiver-mediated intervention requires personal (e.g., time commitment to attend learning/coaching sessions and practice in natural contexts throughout the day) and familial resources (e.g., balancing other priorities) to engage fully. Thus, it may be important for families to experience some tangible concerns about their baby's development first. Indeed, it remains to be fully examined whether participation in a CMI, in the absence of identifiable behavioral signs of autism in the baby, may lead to unnecessary stressors for families. In collaboration with families, this is an area to consider for future research so that we can understand what is considered both timely and helpful.

Finally, although not the focus of this program, encouraging caregivers to pause to support other areas of development (e.g., motor skills) that arise during sessions ensures that we are not interfering with a child's natural growth, an approach that aligns with points raised by Grzadzinski et al. (26), who suggest that these considerations likely result in more positive long-term developmental outcomes.

### 4.1 Limitations

Bradshaw et al. (42) discussed the high rate of families declining to participate in very early intervention programs, finding that socioeconomic factors influenced families’ decisions to attend caregiver-mediated programs and that their basic needs often had to take priority. This finding is consistent with previous work by Stahmer et al. (40), who demonstrated that low-income and ethnic minority families face substantial barriers related to funding and access to care. Future work should consider active recruitment of families from diverse socioeconomic backgrounds.

Enrolment in this study was low, and all the participating caregivers had high levels of education, income, housing, and job and food security. As such, the current work does not allow us to draw conclusions about how a diverse range of families would respond to the program, and we are unable to ascertain how socioeconomic barriers may impact caregivers’ enrolment, acceptance, and response to the Baby Social ABCs.

## 5 Conclusions and future directions

The pilot study results provide preliminary support for the Baby Social ABCs intervention. The findings from this pilot study have allowed us to finalize the Baby Social ABCs program curriculum and develop an enhanced coding scheme that will be used in a larger study. The next steps include finalizing a comprehensive caregiver coaching manual for the baby version and, in preparation for a powered RCT, using the current findings to fine-tune the session protocol.

## Data Availability

The datasets presented in this article are not readily available because this is in compliance with our Research Ethics Board Approval. Requests to access the datasets are not applicable.
